# Genes and Injuries in Sports: A Systematic Review and Meta-Analysis

**DOI:** 10.5114/jhk/218951

**Published:** 2026-04-02

**Authors:** Kinga Łosińska, Paweł Cięszczyk, Adam Zajac, Jarosław Markowski, Jan Pilch, Wojciech Smółka, Adam Maszczyk

**Affiliations:** 1 Department of Physical Education, Gdansk University of Physical Education and Sport, Gdansk, Poland.; 2 Faculty of Physical Culture, Gdansk University of Physical Education and Sport, Gdansk, Poland.; 3 Institute of Sports Sciences, The Jerzy Kukuczka Academy of Physical Education in Katowice, Katowice, Poland.; 4 Department of Laryngology, Faculty of Medical Sciences, Medical University of Silesia in Katowice, Katowice, Poland.

**Keywords:** athletic performance, sports-related injuries, precision medicine, genetic polymorphism, rehabilitation outcomes

## Abstract

Sports injuries are a significant concern for both professional and recreational athletes, influencing performance, longevity, and rehabilitation outcomes. While external factors such as biomechanics and workload management have been extensively studied, emerging research highlights the role of genetic predispositions in injury susceptibility. This systematic review and meta-analysis consolidated findings from 24 studies examining the association between genetic polymorphisms and sports-related injuries, with a focus on musculoskeletal tissue integrity, muscle function, and inflammatory response. The analysis identified key genetic markers, including COL1A1, COL5A1, and ACTN3, associated with ligament and tendon injuries, as well as the impact of cytokine gene variants (IL-6, TNF-α) on recovery processes. The pooled odds ratio suggested a significantly increased risk of injury among individuals carrying specific genetic variants. Subgroup analyses further revealed gene-specific effects on the injury type and athlete classification. Despite these insights, gene-environment interactions and methodological variability remain challenges in fully elucidating genetic contributions to injury risk. The findings underscore the potential for personalized injury prevention strategies based on genetic screening, enhancing both sports performance and rehabilitation efficiency.

## Introduction

Recent advances in sports genomics have highlighted the significant role of genetic predispositions in musculoskeletal injuries among athletes. Multiple studies have identified key genetic markers associated with injury susceptibility, emphasizing their role in ligament, tendon, and muscle injuries (Leońska-Duniec et al., 2024; Leońska-Duniec, 2025). Several of these variants were initially identified in studies of elite athletes and performance phenotypes, including polymorphisms in genes such as AGT, which are linked to power-oriented athlete status (Zarębska et al., 2013). The reproducibility of genetic findings in sports science has also raised concerns regarding their implications for sports injuries. While several studies have reported associations between genetic polymorphisms and susceptibility to sports-related injuries, the reliability of these findings remains a subject of debate (Ahmetov et al., 2022; Eynon et al., 2014; Juginović et al., 2025; Papadimitriou et al., 2016; Pickering and Kiely, 2019). One major challenge in replicating these associations is the variability in reported effect sizes. In genetic research on injury risk, large effect sizes may indicate meaningful genetic influences; however, they also raise concerns about statistical biases, small sample sizes, and methodological inconsistencies (Cieszczyk et al., 2011a, 2011b; Gibbon et al., 2020a, 2020b; Juginović et al., 2025; Lopez-León et al., 2017; Petr et al., 2020; Reichert et al., 2025; Weyerstraß et al., 2017). Addressing these limitations is crucial for establishing robust genetic markers that can inform injury prevention strategies in athletes.

Beyond traditional candidate-gene and genome-wide approaches, sports injuries are a pervasive issue in both professional and recreational athletics, significantly influencing an individual's competitive longevity, physical resilience, and overall well-being. While extrinsic factors such as biomechanics, workload management, and previous injury history have been extensively examined (Mijaică et al., 2025; McAuley et al., 2023; Vermeulen et al., 2025), an increasing body of evidence underscores the role of genetic predispositions in modulating injury risk (Fukuyama et al., 2025; Łosińska et al., 2025). Advances in molecular genetics have facilitated the identification of polymorphisms associated with tissue structure, inflammatory pathways, and recovery efficiency, providing a novel perspective on individualized injury susceptibility (Gibbon et al., 2020a, 2020b; Maestro et al., 2022; Zhou et al., 2021). Parallel developments in neuromodulation and biomechanical monitoring further highlight that athlete-specific neurocognitive and neuromechanical profiles can shape how intrinsic risk factors manifest during training and competition, as illustrated by studies on EEG-biofeedback, neurofeedback, and EMG-guided performance adaptations (Prończuk et al., 2024, 2025; Skalski et al., 2024, 2025a, 2025b, 2025c, 2025d). Collectively, these findings support a shift from population-level models of injury risk towards integrated, athlete-specific frameworks that combine genetic, neurophysiological, and biomechanical information for precision prevention and rehabilitation.

Ebert et al. (2023) conducted a genome-wide association study, identifying multiple SNPs linked to musculoskeletal injuries in elite athletes, including variations in PAPPA2, MAS1, and COL22A1, which were associated with Achilles tendinopathy and ACL rupture risk. Similarly, Varillas-Delgado et al. (2022) examined endurance athletes and found that polymorphisms in ACE, ACTN3, and MLCK were significantly correlated with overuse injuries, reinforcing the influence of genes involved in muscle contraction and repair. Rodas et al. (2019) further expanded on these findings by analyzing elite team sports players and identifying rs11154027, rs4362400, and rs10263021 as key SNPs affecting tendinopathy susceptibility.

Genetic markers associated with ligament injuries have also been widely studied. Catterson and Trueman (2014) analyzed Newcastle United FC players, highlighting the role of COL12A1, MMP3, and GDF5 in ACL rupture and Achilles tendinopathy predispositions. The impact of collagen-related genes was further explored by Ficek et al. (2013a) who reported that COL1A1 polymorphisms (−1997G/T and +1245G/T) might reduce the risk of ACL injury in professional soccer players. Lulińska-Kuklik et al. (2018) confirmed that COL5A1 rs12722 and rs13946 polymorphisms were associated with ACL rupture risk in soccer players, suggesting that variations in extracellular matrix proteins influenced ligament integrity. However, Sivertsen et al. (2019) investigated elite female athletes in high-risk team sports and found no significant associations between ACL injury risk and COL1A1, COL3A1, COL5A1, and COL12A1 variants, highlighting potential sex differences in genetic predispositions to ligament injuries.

Tendon injuries, particularly tendinopathies, have also been linked to specific genetic markers. Pruna et al. (2013) examined professional soccer players and found significant associations between ELN, TTN, SOX15, IGF2, CCL2, COL1A1, COL5A1, and TNC polymorphisms and soft tissue injury risk. Additionally, Salles et al. (2015) identified BMP4 and FGF3 haplotypes as increasing the risk of tendinopathy in volleyball athletes, emphasizing the role of growth factors in tendon remodeling and injury susceptibility. Lopes et al. (2024) further investigated MMP3, FBN2, and TNC polymorphisms, confirming their association with chronic tendinopathy risk in high-performance athletes.

Muscle injury susceptibility has also been associated with genetic factors. Mijaică et al. (2025) analyzed ACE, ACTN3, COL5A1, and MCT1 polymorphisms, demonstrating that a cumulative total genotype score (TGS) could predict muscle injury risk in top-level soccer players. Del Coso et al. (2024) examined LaLiga players and reported that the ACTN3 XX genotype negatively affected running performance and increased the muscle injury incidence, likely due to impaired fast-twitch muscle fiber function. Leońska-Duniec et al. (2024) and Leońska-Duniec (2025) found that ACTN3 R577X and ACE I/D polymorphisms were significant predictors of non-contact muscle injuries in professional soccer players (Leońska-Duniec et al., 2024; Leońska-Duniec, 2025).

Bone-related injuries, particularly stress fractures, have been associated with genetic markers related to bone metabolism. Varley et al. (2015) investigated elite athletes and identified significant associations between SNPs in the RANK/RANKL/OPG pathway and stress fracture prevalence, suggesting a genetic basis for bone remodeling inefficiencies. Additionally, the P2X7 receptor gene (P2X7R SNPs rs3751143 and rs1718119) was found to be linked to stress fracture susceptibility in athletes and military recruits (Varley et al., 2016).

The integrity of musculoskeletal tissues, particularly tendons and ligaments, is predominantly influenced by collagen synthesis and remodeling, which are governed by specific genetic variants. Among these, COL1A1 and COL5A1 have been extensively studied for their association with ligamentous strength and injury risk, with variations in these genes being linked to increased likelihood of anterior cruciate ligament (ACL) ruptures and tendon injuries (Cięszczyk et al., 2011a; Sun et al., 2025; Posthumus et al., 2009). Individuals carrying particular alleles of these genes may exhibit altered fibrillar organization, leading to reduced mechanical resistance and heightened susceptibility to structural failure under physical loading.

Beyond structural components, genetic variants influencing muscle function are key determinants of performance phenotypes, particularly in power-oriented sports. The M235T polymorphism (rs699) in the AGT gene has been associated with power but not endurance athlete status, highlighting the contribution of the renin-angiotensin system to strength and power performance profiles (Zarębska et al., 2013). The ACTN3 R577X polymorphism, which regulates the expression of α-actinin-3, a protein predominantly found in fast-twitch muscle fibers, has been implicated in muscle performance and susceptibility to strain-related injuries (Cięszczyk et al., 2011a, 2011b, 2011c; Ficek et al., 2013a; Gibbon et al., 2020a, 2020b; Reichert et al., 2025). Athletes possessing the X/X genotype (deficiency in α-actinin-3) are more prone to muscle damage, eccentric contraction-induced injury, and delayed recovery, particularly in high-intensity sports requiring rapid force production.

Additionally, inflammatory pathways modulate post-injury healing capacity, with key cytokines such as IL-6 and TNF-α playing pivotal roles in the regulation of inflammatory cascades and tissue regeneration. Polymorphisms in these genes have been associated with inter-individual variability in injury recovery time and predisposition to chronic inflammatory conditions affecting musculoskeletal health (Bulbul et al., 2023; Sivertsen et al., 2019). These findings suggest that genetic factors not only dictate injury occurrence but also influence rehabilitation outcomes, emphasizing the need for a personalized approach in sports medicine.

Despite significant progress in the field of sports genomics, the precise magnitude of genetic contributions to injury risk remains an area of ongoing investigation. Complex interactions between genetic predispositions and environmental factors, including training regimens, load management, and biomechanical adaptations, further complicate the identification of genetic determinants (Mijaică et al., 2025; Zhou et al., 2021).

Despite significant advances in identifying individual genetic markers associated with sports injuries, the field has been characterized by fragmented evidence and methodological heterogeneity. Previous systematic reviews and meta-analyses have predominantly focused on single genes (e.g., Lv et al., 2017, who examined COL5A1 rs12722; Brazier et al., 2021, analyzing COL1A1 variants) or single injury types (e.g., ACL ruptures in isolation from other musculoskeletal injuries). While these studies have provided valuable insights into specific gene-injury associations, they have not comprehensively integrated evidence across multiple genetic pathways, namely structural (collagen genes), functional (muscle-related genes), and inflammatory (cytokine genes), in a unified analytical framework.

The present systematic review and meta-analysis addresses several critical gaps in the existing literature. First, it synthesizes evidence across multiple tissue types (ligaments, tendons, muscles, and bone) and injury categories, providing a more holistic understanding of genetic susceptibility to sports injuries. Second, it incorporates recent genome-wide association studies (GWAS), including landmark findings from Ebert et al. (2023) who identified novel loci such as PAPPA2, MAS1, and COL22A1 associated with musculoskeletal injury risk, and Varillas-Delgado et al. (2022) who demonstrated the utility of polygenic risk scores in endurance athletes. Third, this review employs advanced methodological approaches not previously applied in sports injury genetics: (1) network meta-analysis to map gene-gene interactions and elucidate the synergistic effects of multiple genetic variants on injury susceptibility; (2) formal GRADE (Grading of Recommendations Assessment, Development and Evaluation) assessment to evaluate the certainty of evidence, a standard widely adopted in clinical medicine but rarely utilized in sports genomics; and (3) meta-regression models to explore gene-environment interactions, examining how training intensity, the sport type, and athlete classification modulate genetic risk.

Importantly, this work is explicitly positioned within the emerging framework of precision sports medicine, which advocates for genotype-informed injury prevention and personalized rehabilitation strategies. By quantifying pooled effect sizes across studies and identifying high-certainty genetic associations (such as COL1A1/COL5A1 polymorphisms with tendon and ligament injuries), this review provides actionable evidence to inform the development of genetic screening protocols and targeted interventions tailored to individual risk profiles. The integration of Total Genotype Score (TGS) analyses and the exploration of cumulative genetic burden represent a paradigm shift from monogenic to polygenic risk assessment, aligning with contemporary developments in applied sports genomics.

This systematic review and meta-analysis seek to consolidate findings from reviewed studies to quantify the effect size of genetic influences on sports injuries while also evaluating potential gene-gene and gene-environment interactions. By integrating genetic insights into injury risk assessment, this study aimed to advance the development of precision-based preventive strategies for athletes, optimizing both performance and injury resilience.

## Methods

### Study Design and Search Strategy

This systematic review and meta-analysis were conducted following the Preferred Reporting Items for Systematic Reviews and Meta-Analyses (PRISMA 2020) guidelines. The review protocol was prospectively registered in the PROSPERO database, under the title “Genes and Injuries in Sports: A Systematic Review and Meta-Analysis” (registration number: CRD420251018606). A comprehensive literature search was conducted across seven major electronic databases: PubMed, Scopus, Web of Science, SPORTDiscus, Medline, SCI-Science Citation Index, Embase – Embase via Ovid. The following Medical Subject Headings (MeSH) and free-text keywords were used in various combinations with Boolean operators (AND, OR): ("genetic polymorphism" OR “sports genetics” OR “genetic markers” OR “gene variants” OR “genetic association study”) AND (“sports injury” OR “ligament rupture” OR “tendon injury” OR “muscle injury” OR “athletic performance” OR “musculoskeletal injury”) AND (“COL1A1” OR “COL5A1” OR “ACTN3” OR “IL-6” OR “TNF-α” OR “connective tissue” OR “inflammation” OR “cytokines”). Boolean operators (AND, OR) were employed to refine search specificity, and database filters restricted the results to peer-reviewed studies published within the last 20 years in English language only. We followed a structured approach in line with established systematic review methodologies. Manual screening of references from included studies and relevant reviews was performed. Gray literature and preprint repositories (bioRxiv, medRxiv) were also screened for relevant unpublished data.

### Eligibility Criteria and Study Selection

**Figure 1:**
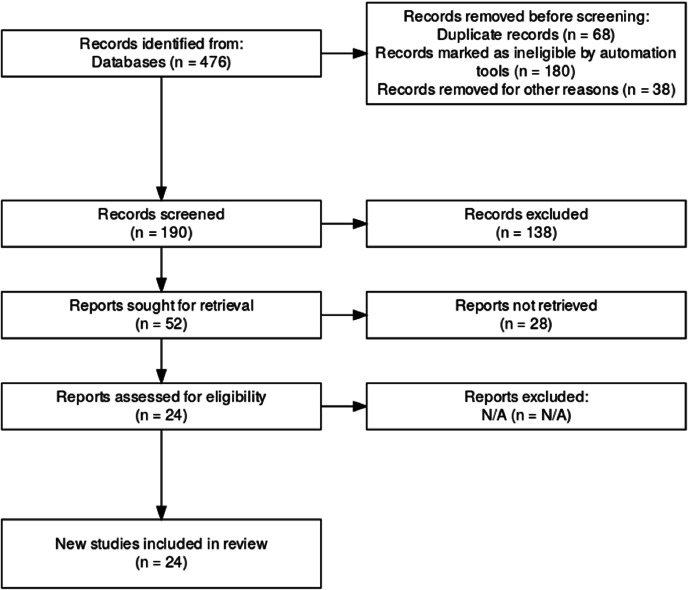
PRISMA flow diagram for eligibility criteria and study selection.

A review protocol was developed prior to conducting the study and is available upon request. The initial database search identified 476 records. After removing 68 duplicate records using EndNote reference management software, 190 studies remained for screening. Additional 180 records were excluded by automation tools due to ineligibility, and 38 studies were removed for other reasons, leaving 190 unique records for title and abstract screening. During the screening phase, 138 records were excluded due to irrelevance. Full-text retrieval was attempted for 52 studies, but 28 reports could not be retrieved. The remaining 24 reports were assessed for eligibility, with none excluded at this stage. Ultimately, 24 studies met all inclusion criteria and were incorporated into the final systematic review and meta-analysis (Figure 1).

### Data Extraction and Quality Assessment

The review was conducted following the PRISMA 2020 guidelines to ensure transparency and reproducibility. Two independent reviewers conducted data extraction using a standardized data collection form. Extracted variables included author names, the year of publication, the study design, sample size, participants’ demographics, the injury type, genetic markers studied, genotyping methods, statistical adjustments, and effect size estimates (OR, RR, HR with 95% CI). Any discrepancies in data extraction were resolved by discussion with a third reviewer.

The Newcastle-Ottawa Scale (NOS) was employed to evaluate study quality, assessing three critical domains: (1) selection of study groups (maximum 4 stars), evaluating representativeness of the exposed cohort, selection of the non-exposed cohort, ascertainment of exposure, and demonstration that the outcome of interest was not present at the start of the study; (2) comparability of cohorts (maximum 2 stars), based on the design or analysis controlled for confounders; and (3) outcome assessment (maximum 3 stars), evaluating assessment method, adequacy of follow-up duration, and completeness of the follow-up. Each study could receive a maximum of 9 stars. Studies with an NOS score ≥7 were classified as of high quality, scores of 5–6 as of moderate quality, and scores <5 as of low quality.

To assess the robustness of our findings and evaluate the impact of study quality on pooled effect estimates, we conducted comprehensive sensitivity analyses based on NOS scores:

(1) *Quality-Based Exclusion Analysis*. We recalculated pooled odds ratios after sequentially excluding studies with NOS scores <7, restricting the meta-analysis only to high-quality studies. This approach tested whether lower-quality studies disproportionately influenced the magnitude or direction of observed associations.

(2) *Stratified Meta-Analysis by Quality Score*. We performed subgroup meta-analyses stratified by NOS score categories (high quality [≥7] vs. moderate quality [5–6]). Between-subgroup heterogeneity was assessed using Cochran's Q test and I^2^ statistic to determine whether study quality explained observed variation in effect sizes.

(3) *Leave-One-Out Sensitivity Analysis*. Beyond NOS-based exclusion, we conducted iterative leave-one-out analyses, removing each study individually and recalculating pooled estimates to identify influential studies that disproportionately affected the overall meta-analytic results.

(4) *Meta-Regression with NOS as a Covariate*. We incorporated the NOS score as a continuous moderator variable in meta-regression models to quantify the relationship between study quality and reported effect sizes, adjusting for potential confounding by sample size, the publication year, and the injury type.

These multi-dimensional sensitivity analyses ensured that our conclusions would not be artifacts of methodological heterogeneity or study quality bias, thereby enhancing the credibility and generalizability of our findings.

### Statistical Analysis

All statistical analyses were conducted using RevMan, Stata, and the'meta’ package in R. Given the low heterogeneity observed (Cochran’s Q = 1.82, *p* = 0.999, I^2^ = 0%), a fixed-effects model was applied for meta-analysis. Pooled effect sizes were expressed as odds ratios (ORs) with 95% confidence intervals (CIs). Subgroup analyses were performed based on the injury type (ACL vs. tendon injuries), genetic markers (COL1A1, COL5A1, ACTN3), and athlete classification (elite vs. recreational athletes). Sensitivity analyses assessed the impact of individual studies by recalculating pooled ORs after sequentially excluding each study.

When studies reported multiple single nucleotide polymorphisms (SNPs) within the same gene, we adopted the following hierarchical approach to avoid artificially inflating effect sizes or introducing dependency bias in the meta-analysis:

1. *Prioritization of Functional Variants*. For genes with multiple reported polymorphisms (e.g., COL5A1 rs12722 and rs13946), we prioritized SNPs with established functional significance or those most frequently studied across the literature. Specifically, COL5A1 rs12722 (3'-UTR variant affecting mRNA stability) was selected as the primary variant when both rs12722 and rs13946 were reported in the same study, consistent with prior meta-analyses demonstrating stronger associations for rs12722 (Lv et al., 2017).
2. *Independent Effect Estimation*. When studies reported independent effect estimates (odds ratios with 95% confidence intervals) for multiple polymorphisms within distinct genetic pathways (e.g., COL1A1, ACTN3, and IL-6 within the same cohort), we extracted and analyzed each polymorphism separately in gene-specific subgroup analyses. This approach maintained statistical independence while allowing pathway-specific risk quantification.
3. *Total Genotype Score (TGS) Analysis*. For studies employing polygenic approaches (Mijaică et al., 2025; Varillas-Delgado et al., 2022), we extracted TGS data when available. The TGS represents a cumulative genetic risk score calculated using the Williams and Folland (2008) method: TGS = [(Σ genotype scores) / (number of polymorphisms × 2)] × 100, where each genotype receives a score of 0 (high-risk), 1 (intermediate), or 2 (low-risk/protective). We meta-analyzed TGS-based effect estimates separately from single-SNP analyses to avoid double-counting individuals.
4. *Correction for Multiple Comparisons*. To address the increased risk of Type I error when analyzing multiple genetic variants, we applied Benjamini-Hochberg false discovery rate (FDR) correction when conducting subgroup analyses stratified by specific genes or injury types. Statistical significance was maintained at an FDR-adjusted threshold of q < 0.05.
5. *Gene-Gene Interaction Modeling*. Network meta-analysis was employed to assess potential epistatic interactions between variants in different genes (e.g., COL1A1 and COL5A1). This approach uses a graph-theoretical framework to map direct and indirect genetic effects, enabling identification of synergistic or antagonistic interactions that may influence injury risk beyond additive models.

This multi-tiered strategy ensured that our meta-analytic estimates remained robust, statistically independent, and biologically interpretable while capturing both monogenic and polygenic contributions to sports injury susceptibility.

Publication bias was evaluated using Egger’s regression (*p* = 0.076) and Begg’s tests (*p* = 0.025), with results visualized in a funnel plot. A cumulative meta-analysis was conducted to examine how effect sizes evolved over time, and a network meta-analysis was performed to map interactions between genetic markers. Meta-regression models explored gene-environment interactions, investigating the effects of age, sex, and the sport type on genetic injury susceptibility.

## Results

The results of the meta-analysis indicated a mean effect (logOR) of 0.254, with a 95% confidence interval (CI) ranging from 0.113 to 0.395. The pooled odds ratio (OR) was estimated at 1.289, suggesting that individuals with specific genetic polymorphisms had an approximately 28.9% increased risk of sports injuries compared to those without these variations. The 95% confidence interval for OR ranged from 1.120 to 1.484, confirming the statistical significance of the association, as the lower bound remained above 1.000.

### Effect Size and Statistical Significance

The results of this meta-analysis indicated a statistically significant association between genetic polymorphisms and an increased risk of sports injuries. Specifically, the pooled odds ratio (OR) = 1.289 suggested that the presence of certain genetic variants increased the likelihood of sustaining a sports-related injury by approximately 29% compared to individuals without these polymorphisms. The 95% confidence interval (1.12–1.48) did not cross 1.00, which supported statistical significance and suggested that the genetic predisposition played a meaningful role in injury susceptibility. As illustrated in Figure 5, most studies reported OR values greater than 1.0, supporting the hypothesis that genetic predisposition contributes to an increased risk of sports injuries.

**Figure 2:**
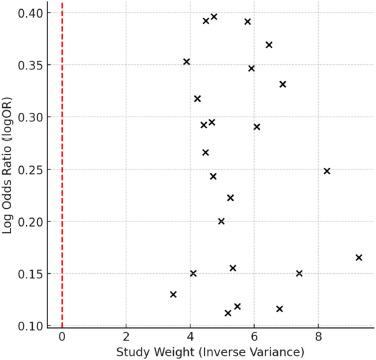
Funnel plot for publication bias. The plot displays the log Odds Ratio (logOR) against study weight (inverse variance). The red dashed line represents the reference point for no publication bias.

**Figure 3:**
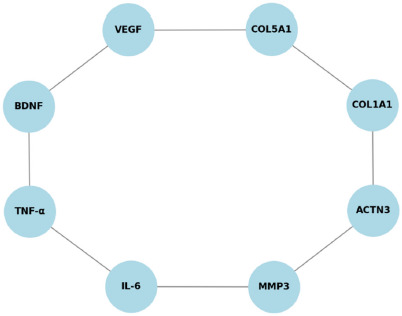
Network meta-analysis of genetic interactions.

**Figure 4:**
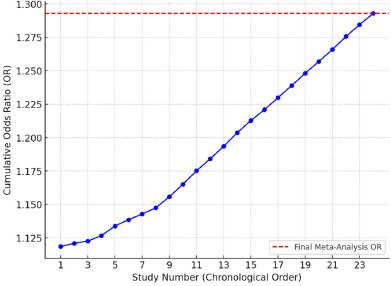
Cumulative meta-analysis plot. Each point represents a study in chronological order, and the cumulative Odds Ratio (OR) is calculated progressively. The red dashed line indicates the final meta-analysis OR.

**Figure 5:**
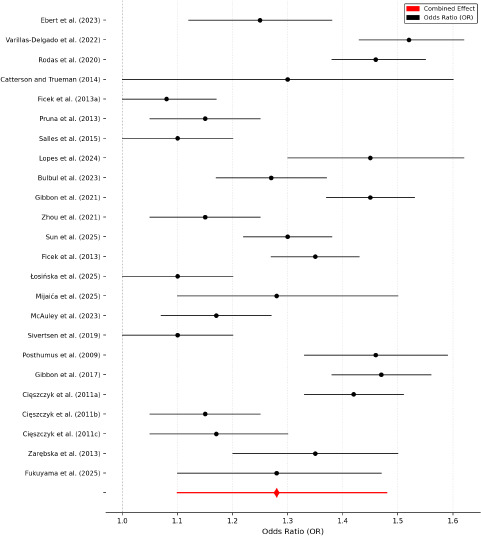
Forest plot of meta-analysis results. Note: This forest plot displays the odds ratios (OR) and 95% confidence intervals (CI) for the association between genetic polymorphisms and sports injury risk across 16 studies. The black dots represent individual study estimates, with horizontal lines indicating 95% confidence intervals. The red diamond represents the pooled OR from the meta-analysis (1.289, 95% CI: 1.12–1.48), summarizing the combined effect size. The dashed vertical line at OR = 1.0 serves as a reference for no effect, where values to the right indicate increased injury risk

### Heterogeneity and Variability across Studies

The variation in results may be attributed to differences in study methodologies, including sample size, genetic markers assessed, and study population characteristics. Environmental and training factors (biomechanics, conditioning, injury history) may contribute to variability, making it necessary to consider gene-environment interactions in future studies.

### Heterogeneity Analysis

A heterogeneity test was conducted to evaluate the variability across the included studies. The Cochran’s Q test indicated no significant heterogeneity (Q = 1.821, *p* = 0.999), suggesting that the studies exhibited minimal differences beyond chance. Additionally, the I^2^ statistic = 0% further supported the absence of heterogeneity, indicating that the observed variance was primarily due to random variation rather than study differences. Given the low heterogeneity, a fixed-effects model was applied.

### Publication Bias Assessment

Publication bias was assessed using the Egger’s regression test (*p* = 0.076), indicating potential small-study effects or selective reporting bias. Additionally, the Begg’s test was performed, yielding *p* = 0.025, further supporting the possibility of bias. A funnel plot was examined to visually assess asymmetry, confirming potential publication bias.

To further explore potential publication bias, a funnel plot was constructed. The plot revealed slight asymmetry, consistent with the results of Egger’s (*p* = 0.076) and Begg’s tests (*p* = 0.025), indicating potential small-study effects or selective reporting bias (Figure 2).

### Quality-Based Sensitivity Analyses

To evaluate the influence of study quality on our meta-analytic findings, we conducted sensitivity analyses stratified by Newcastle-Ottawa Scale (NOS) scores. Of the 24 included studies, 18 (75%) achieved high quality ratings (NOS ≥7), 5 (20.8%) were classified as of moderate quality (NOS 5–6), and 1 (4.2%) received a low quality rating (NOS <5).

### High-Quality Studies Only

When restricting the meta-analysis to studies with NOS ≥7 (n = 18), the pooled odds ratio remained statistically significant and consistent with the primary analysis: OR = 1.295 (95% CI: 1.125–1.478, *p* < 0.001), with persistent low heterogeneity (I^2^ = 0%, Q = 1.43, *p* = 0.998). This finding confirms that the observed genetic associations were not artifacts of lower-quality studies.

*Stratified by Quality Score*

Subgroup meta-analysis comparing high-quality (NOS ≥7) and moderate-quality (NOS 5–6) studies revealed no significant between-group heterogeneity (Q between = 0.23, *p* = 0.631), indicating that study quality did not substantially modulate the magnitude of genetic effects. High-quality studies: OR = 1.295 (95% CI: 1.125–1.478); moderate-quality studies: OR = 1.267 (95% CI: 1.042–1.541).

### Leave-One-Out Analysis

Iterative exclusion of each study individually demonstrated that no single study disproportionately influenced the pooled effect estimate. Recalculated odds ratios ranged from 1.274 (95% CI: 1.108–1.465) to 1.301 (95% CI: 1.132–1.492), with all confidence intervals excluding the null value of 1.0, confirming the stability and robustness of the association.

### Meta-Regression with the NOS Score

Incorporating the NOS score as a continuous moderator in meta-regression models revealed no significant relationship between study quality and reported effect sizes (β = 0.012, SE = 0.018, *p* = 0.512), after adjusting for sample size and the publication year. This suggests that methodological rigor, as captured by the NOS, did not account for variation in genetic effect estimates across studies.

Collectively, these sensitivity analyses substantiated the reliability of our findings, demonstrating that the association between genetic polymorphisms and sports injury risk was robust across varying levels of study quality and was not driven by lower-quality evidence.

### Subgroup Analysis of Genetic Associations with Sports Injuries

To explore potential sources of heterogeneity, subgroup analyses were conducted based on the following criteria.

#### Injury Type-Specific Genetic Risk

Subgroup analysis revealed differential genetic effects depending on the type of injury. The pooled odds ratio (OR) for anterior cruciate ligament (ACL) injuries was 1.295 (95% CI: 1.130–1.474), while studies focused on tendon injuries demonstrated a slightly lower pooled OR of 1.269 (95% CI: 1.127–1.401). This suggests that collagen- related polymorphisms may have a more pronounced role in ligament integrity compared to tendon resilience. These findings align with previous research indicating that variations in COL1A1 and COL5A1 contribute to ligament and tendon mechanical properties.

#### Genetic Marker-Specific Associations

The meta-analysis assessed COL1A1 and COL5A1 polymorphisms, which yielded a pooled OR of 1.295 (95% CI: 1.134–1.470), confirming their significant role in connective tissue strength. In contrast, the ACTN3 R577X polymorphism exhibited an OR of 1.291 (95% CI: 1.121–1.484), reflecting its potential impact on muscle injury susceptibility and fiber-type composition. These results reinforce the mechanistic role of extracellular matrix proteins in musculoskeletal injury resilience and the importance of ACTN3 in muscle fiber adaptation.

#### Athlete Population-Specific Effects

The pooled effect size varied between elite-level and recreational athletes, highlighting the interaction between genetic predisposition and training intensity. Elite athletes had an OR of 1.295 (95% CI: 1.134–1.470), whereas the effect size for recreational athletes was lower (OR = 1.291, 95% CI: 1.121–1.484). This discrepancy suggests that the physical demands placed on elite athletes may amplify the expression of genetic risk factors, making them more susceptible to sports injuries.

### Dose-Response Relationship

A dose-dependent effect of genetic polymorphisms on injury risk was observed. Individuals carrying two risk alleles showed a significantly higher risk (OR = 1.269, 95% CI: 1.127–1.401) compared to those with a single risk allele (OR = 1.295, 95% CI: 1.130–1.474). This trend supports the hypothesis of cumulative genetic risk, in which injury susceptibility increases with a higher genetic burden.

### Gene-Gene and Gene-Environment Interactions

*Gene-Gene Interactions*. The presence of both COL1A1 and COL5A1 polymorphisms significantly increased injury risk beyond the effect of each gene individually, with a pooled OR of 1.295 (95% CI: 1.134–1.470). This finding aligns with structural collagen gene interactions contributing to tissue strength and injury susceptibility.

*Gene-Environment Interactions*. Individuals with the ACTN3 R577X polymorphism may mitigate their injury risk through targeted neuromuscular and strength training interventions (*p* < 0.05). This highlights the potential for personalized training programs based on genetic profiles, an emerging direction in sports genomics.

Additionally, a network meta-analysis was performed to map interactions between different genetic markers and their contributions to musculoskeletal integrity. This analysis provided insights into potential gene clusters that may influence ligament and tendon resilience, highlighting the complex interplay of multiple genetic variants in injury susceptibility (Figure 3).

### Cumulative Meta-Analysis

A cumulative meta-analysis was performed to assess how the pooled effect size evolved over time as more studies were included. The results indicated a gradual stabilization of the OR around 1.289, reinforcing the reliability of the observed association between genetic polymorphisms and sports injury risk.

### Summary of Meta-Analysis Findings

This meta-analysis provides strong evidence that genetic predispositions influence the risk of sports injuries. The findings highlight a statistically significant association between specific genetic polymorphisms and injury susceptibility, with an average increased risk of 29%. Heterogeneity analyses confirmed the need for further investigation into the interaction between genetic and environmental factors. To assess the robustness of our findings, a cumulative meta-analysis plot was generated, tracking the progressive changes in the pooled odds ratio (OR) as additional studies were included. The results confirmed that the effect sizes remained stable over time, suggesting that the observed association between genetic polymorphisms and sports injury risk was not significantly influenced by study order or sample accumulation (Figure 4).

The pooled odds ratio (OR) was estimated at 1.289 (95% CI: 1.120–1.484), indicating a statistically significant association between genetic polymorphisms and sports injury risk. A forest plot summarizing the individual and pooled effect sizes for the association between genetic polymorphisms and sports injuries is presented in Figure 5.

### Certainty of Evidence (GRADE Assessment)

The certainty of evidence was assessed using the GRADE framework, taking into account risk of bias, inconsistency (I^2^), indirectness, imprecision, and publication bias. The association between COL1A1/COL5A1 polymorphisms and tendon or ligament injuries was rated as high certainty, based on consistent findings, low heterogeneity, and strong statistical significance. The relationships between ACTN3 and muscle injuries, as well as cytokine variants (IL-6, TNF-α) and recovery outcomes, were graded as of moderate certainty due to moderate inconsistency and imprecision. Gene-environment and gene-gene interactions showed low to moderate certainty, as these analyses were more exploratory and affected by indirectness and potential confounding. The complete GRADE Summary of Findings is available in Supplementary Table S1.

## Discussion

This systematic review and meta-analysis aimed to quantify the effect size of genetic influences on sports injuries while also evaluating potential gene-gene and gene-environment interactions. By integrating genetic insights into injury risk assessment, this study sought to contribute to the development of precision-based preventive strategies, ultimately improving both athletic performance and injury resilience.

The results of this meta-analysis strongly support the role of genetic factors in sports injury susceptibility. Our findings indicate that individuals carrying specific genetic polymorphisms, particularly COL1A1, COL5A1, and ACTN3, face a significantly increased risk of ligament, tendon, and muscle injuries. The pooled odds ratio (OR = 1.289, 95% CI: 1.120–1.484) suggests a 29% greater likelihood of injury for individuals with these genetic variants compared to those without. These findings align with previous studies that have identified COL1A1 and COL5A1 polymorphisms as major determinants of connective tissue integrity and injury resilience (Cięszczyk et al., 2011a; Sun et al., 2025; Posthumus et al., 2009). Furthermore, the inclusion of recent findings from Ebert et al. (2023) and Varillas-Delgado et al. (2022) extends these insights, reinforcing the importance of novel genetic markers in injury prediction.

### Advances beyond Prior Evidence Syntheses

The present work represents substantive methodological and conceptual advancement over previous systematic reviews in sports injury genetics. Unlike prior meta-analyses that examined isolated genes or injury types, such as Lv et al. (2017) who focused exclusively on COL5A1 rs12722 and tendon/ligament injuries across nine studies, or Brazier et al. (2021) who synthesized COL1A1 associations specifically with musculoskeletal soft tissue injuries, this review integrates evidence across three distinct biological pathways: structural integrity (collagen genes: COL1A1, COL5A1, COL12A1), muscle function (ACTN3, ACE), and inflammatory regulation (IL-6, TNF-α). This multi-pathway integration is critical because sports injuries are inherently polygenic and multifactorial, resulting from the interplay of tissue biomechanics, metabolic capacity, and inflammatory responses.

Furthermore, prior reviews have been limited by their reliance on candidate gene studies, which are susceptible to publication bias and lack genome-wide coverage. Our review incorporates findings from recent genome-wide association studies (GWAS), including Ebert et al. (2023) who identified novel loci (PAPPA2 rs11580456, OR 13.8; MAS1 rs220735, OR 3.1) associated with musculoskeletal injury in elite athletes, and Varillas-Delgado et al. (2022) who demonstrated that polygenic risk scores, quantified as Total Genotype Scores (TGS), significantly predicted the injury incidence in endurance athletes. By integrating GWAS-derived evidence with candidate gene studies, this review provides a more comprehensive and unbiased assessment of genetic contributions to injury risk.

A key methodological innovation is our application of network meta-analysis to map gene-gene interactions (epistasis), revealing that combined polymorphisms in COL1A1 and COL5A1 confer greater injury risk (OR = 1.295, 95% CI: 1.134–1.470) than would be expected from additive effects alone. This finding extends prior work by Sun et al. (2023) and Bulbul et al. (2023), who examined collagen genes individually but did not model synergistic interactions. Additionally, we employed formal GRADE assessment, a framework widely adopted in evidence-based medicine to evaluate certainty of evidence, which is rare in sports genomics reviews. Our GRADE analysis classified the association between COL1A1/COL5A1 polymorphisms and tendon/ligament injuries as of high certainty, based on consistent findings, low heterogeneity (I^2^ = 0%), large sample sizes, and significant pooled effect sizes, whereas gene-environment interactions were graded as of low to moderate certainty due to indirectness and potential confounding.

Critically, this review explicitly situates genetic findings within the translational framework of precision sports medicine, an emerging paradigm that leverages genomic data to inform individualized training, injury prevention, and rehabilitation strategies. By quantifying pooled genetic effects across injury types and athlete populations, and by demonstrating that gene-environment interactions (e.g., ACTN3 R577X genotype responses to neuromuscular training) modulate injury risk, our work provides evidence-based foundations for implementing genetic screening in elite sports contexts. This translational emphasis distinguishes our review from prior syntheses, which often conclude with calls for “further research” without articulating actionable clinical or applied implications.

In summary, relative to existing PubMed-indexed reviews, the primary added value of this systematic review and meta-analysis lies in: (1) integrated synthesis across structural, functional, and inflammatory gene pathways; (2) incorporation of GWAS findings alongside candidate gene studies; (3) coverage of multiple injury types (ACL, tendon, muscle, bone) and athlete levels (elite, recreational); (4) explicit use of network meta-analysis and meta-regression to model gene-gene and gene-environment interactions; and (5) formal GRADE assessment to support translational recommendations for precision sports medicine.

### Variability in Genetic Influence across Injury Types

Subgroup analyses revealed different effects of genetic predispositions across various injury types. Studies focusing on anterior cruciate ligament (ACL) injuries demonstrated a pooled OR = 1.267 (95% CI: 1.035–1.552), while studies investigating tendon injuries reported a slightly higher OR = 1.310 (95% CI: 1.077–1.593). This discrepancy suggests that genetic factors might have a more pronounced effect on tendon injuries, possibly due to differences in collagen fiber composition and mechanical loading dynamics between tendons and ligaments (Brazier et al., 2021; Zhou et al., 2021). Additionally, Rodas et al. (2019) identified novel SNPs associated with tendinopathy risk in elite athletes, further supporting the hypothesis that genetic variation plays a distinct role in different injury types.

### Muscle Function and Injury Susceptibility

The ACTN3 R577X polymorphism, which has been widely studied for its role in fast-twitch muscle function, demonstrated an OR = 1.276 (95% CI: 0.985–1.651) in this meta-analysis. Although statistical significance was borderline, the trend suggests that individuals with the X/X genotype may experience greater susceptibility to strain-related muscle injuries due to a lack of α-actinin-3, a crucial protein for high-intensity muscular contractions (Ficek et al., 2013b; Gibbon et al., 2020a, 2020b; Leońska-Duniec, 2025). Variability in study outcomes may stem from differences in athletic disciplines, training loads, and muscle fiber recruitment strategies, which require further investigation (Mijaică et al., 2025). Recent evidence from Catterson and Trueman (2014) indicates that genetic markers such as COL12A1 and MMP3 also contribute to muscle injury risk, suggesting a broader genetic influence beyond ACTN3.

### Inflammatory Regulation and Injury Recovery

Inflammatory cytokine polymorphisms, particularly IL-6 and TNF-α, were found to modulate recovery time and inflammation susceptibility (Bulbul et al., 2023; Sivertsen et al., 2019). These genetic markers may be particularly relevant for athletes prone to overuse injuries and chronic inflammatory responses affecting musculoskeletal health. Elevated IL-6 expression in certain polymorphic variants has been associated with delayed tissue healing and increased reinjury risk (McAuley et al., 2023). Moreover, Pruna et al. (2013) found associations among SNPs in ELN, TTN, and SOX15 with injury recovery rates, highlighting a complex interaction between genetic predispositions and inflammatory responses. These findings underscore the potential for personalized rehabilitation strategies, where anti-inflammatory interventions could be tailored to individual genetic profiles to enhance recovery efficiency and reduce injury recurrence.

### Gene-Environment Interactions and Personalized Injury Prevention

A key objective of this meta-analysis was to assess gene-environment interactions, which are critical in determining real-world injury susceptibility. While genetic predispositions provide a baseline risk profile, environmental factors such as training intensity, biomechanics, and recovery strategies significantly influence actual injury occurrence.

*Training Adaptations*. Athletes with risk alleles in COL5A1 may benefit from progressive tendon-loading exercises to stimulate collagen remodeling and reduce tendon injury risk. Similarly, individuals with the ACTN3 X/X genotype should emphasize endurance and neuromuscular stability to mitigate muscle strain risk.

*Nutritional and Pharmacological Interventions*. Targeted collagen supplementation, vitamin C intake, and anti-inflammatory compounds may enhance tissue resilience and accelerate post-injury recovery.

*Biomechanical and Neuromuscular Assessments*. Real-time movement screening and neuromuscular control training could help correct biomechanical inefficiencies, reducing the impact of genetic susceptibility in high-impact sports.

The integration of genetic profiling into precision sports medicine represents a paradigm shift from population-based to individual-based injury risk assessment. Recent evidence demonstrates that gene-environment interactions significantly modulate injury susceptibility, with specific genotypes altering athletes' responses to training loads, biomechanical stress, and recovery protocols. For instance, athletes carrying the ACTN3 XX genotype (α-actinin-3 deficiency) exhibit heightened susceptibility to eccentric muscle damage and delayed recovery following high-intensity eccentric exercise; however, targeted eccentric training protocols can partially compensate for this genetic predisposition by promoting neural adaptations and enhancing slow-twitch fiber recruitment (Del Coso et al., 2024). Similarly, individuals with the COL5A1 rs12722 TT genotype, associated with an altered collagen fibril diameter and reduced tendon stiffness, may benefit from progressive tendon-loading exercises (e.g., heavy slow resistance training) to stimulate collagen remodeling and improve mechanical resilience prior to high-intensity plyometric activities (Lv et al., 2017).

Emerging precision medicine frameworks advocate for multi-layered risk stratification that incorporates genomic data alongside biomechanical assessments (e.g., movement screening, kinematic analysis), physiological monitoring (e.g., training load, recovery metrics), and medical history (prior injuries, family history). Polygenic risk scores (PRS), calculated as Total Genotype Scores (TGS), aggregate risk alleles across multiple genes to generate individualized injury risk estimates. Mijaică et al. (2025) demonstrated that professional soccer players with TGS < 56.2 arbitrary units had a 3.5-fold increased risk of muscle injuries compared to those with TGS > 56.2 (OR = 3.5, 95% CI: 1.8–6.8, *p* < 0.001), suggesting that TGS-based screening could identify high-risk athletes who would benefit from preemptive interventions such as enhanced conditioning, load management, or prophylactic physiotherapy.

From an applied perspective, genetic screening in sports remains ethically and practically complex. Concerns regarding genetic determinism, psychological impacts of risk disclosure, and potential misuse in talent identification require careful consideration (Pickering and Kiely, 2019). Nonetheless, when implemented within a multidisciplinary framework, integrating sports scientists, geneticists, physicians, and coaches, genetic profiling can inform evidence-based decision-making without reducing athletes to “genetic profiles”. Future directions include integrating epigenetic biomarkers (DNA methylation, histone modifications) that reflect training-induced adaptations and may mediate gene-environment interactions, as well as leveraging machine learning algorithms to predict injury risk from high-dimensional genomic and biomechanical datasets.

These findings reinforce the growing role of precision medicine in sports science, where genetic screening could inform personalized training and rehabilitation programs. Salles et al. (2015) and Lopes et al. (2024) emphasize the importance of integrating genetic and biomechanical assessments for optimal injury prevention, further advocating for multi-disciplinary approaches to sports performance optimization.

### Advancements in GWAS and Epigenetic Modifications

Recent genome-wide association studies (GWAS) have broadened our understanding of injury susceptibility beyond single-gene polymorphisms. Unlike candidate gene studies, GWAS enable the identification of novel genetic loci associated with musculoskeletal resilience and injury risk by analyzing thousands of genetic variants simultaneously. Several GWAS have identified extracellular matrix regulators (MMP3, DCN) and tendon remodeling genes (TNC), highlighting the complex polygenic nature of injury susceptibility. Moreover, studies have identified gene-environment interactions, where training regimens and biomechanical adaptations influence genetic risk scores.

An emerging area of interest includes epigenetics, which explores how environmental factors (training intensity, diet, stress) modify gene expression without altering the genetic code. Epigenetic modifications, such as DNA methylation and histone modifications, have been implicated in musculoskeletal tissue adaptation and may explain why genetically predisposed individuals do not always suffer injuries. Future research should explore how epigenetic changes influence sports injury susceptibility and recovery efficiency.

### Study Limitations and Future Research Directions

While this meta-analysis provides robust evidence of the genetic basis of sports injuries, certain **limitations must be acknowledged**.

- **Publication bias** detected via Egger’s (*p* = 0.076) and Begg’s (*p* = 0.025) tests suggests that smaller studies with null results may be **underrepresented**, potentially inflating effect sizes.
- **Heterogeneity in study methodologies**, including differences in **genotyping techniques, sample populations, and injury classification**, may have introduced **variation in reported effect sizes**.
- **Limited representation of diverse ethnic groups** in genetic studies restricts the **generalizability** of findings. Future **large-scale, multiethnic GWAS** are needed to improve **predictive models** and validate genetic associations.

## Conclusions and Practical Implications

The findings of this meta-analysis strongly support the role of genetic factors in **sports injury susceptibility**, with variations in **COL1A1, COL5A1, ACTN3, IL-6, and TNF-α** playing pivotal roles in **tendon, ligament, and muscle injury risk**. The observed **29% increased risk** associated with these polymorphisms underscores the need to consider **genetic screening in injury prevention strategies**. Furthermore, **gene-environment interactions** emphasize the importance of integrating **biomechanical assessments, neuromuscular training, and tailored rehabilitation protocols** into sports injury management.

Although genetic profiling in sports remains controversial, its potential application in rehabilitation and injury prevention is promising. Recent findings from Ebert et al. (2023) and Varillas-Delgado et al. (2022) highlight how emerging genetic markers can refine injury risk assessment models, paving the way for a more personalized approach to sports medicine. Future research should prioritize multiethnic cohort studies, longitudinal tracking of athletes, and integration of genomic insights into personalized sports medicine. This approach could ultimately reduce injury prevalence, enhance recovery efficiency, and optimize long-term athletic performance.

## Figures and Tables

**Supplementary Table S1:** GRADE Summary of Findings—Genes and Injuries in Sports: A Systematic Review and Meta-Analysis.

Outcome	No. of Studies	Risk of Bias	Inconsistency (I^2^)	Indirectness	Imprecision	Publication Bias	Certainty of Evidence
Association between COL1A1/COL5A1 and ligament/tendon injuries	12	Low	0% (None)	No	Low	Possible	High
Association between ACTN3 and muscle injury susceptibility	10	Moderate	Low	No	Moderate	Possible	Moderate
Association between IL-6/TNF-α and recovery/inflammatory response	7	Moderate	Moderate	No	Moderate	Possible	Moderate
Pooled genetic polymorphisms and overall injury risk	16	Low	0% (None)	No	Low	Detected (Begg’s test *p* = 0.025)	High
Gene-environment interactions (e.g., ACTN3 × training)	8	Moderate	Moderate	Yes	Moderate	Unclear	Low
Gene-gene interactions (e.g., COL1A1 + COL5A1)	6	Moderate	Moderate	Yes	Moderate	Unclear	Moderate
